# Relationship Between Sperm DNA Fragmentation and Composite Morphological Indices: A Multivariable Analysis

**DOI:** 10.3390/medicina62040679

**Published:** 2026-04-02

**Authors:** Veliscu Andreea Carp, Liana Ștefan, Petronela Naghi, Diana Mocuța, Cristina Aur, Liliana Sachelarie, Mircea Sandor

**Affiliations:** 1 Department of Clinical Disciplines, University of Medicine and Pharmacy Carol Davila, Eroii Sanitari Boulevard, 050474 Bucharest, Romania; andreea.veliscu@umfcd.ro; 2 Department of Surgical Disciplines, Faculty of Medicine and Pharmacy, University of Oradea, 10 Piata 1 Decembrie Street, 410073 Oradea, Romania; dmocuta@uoradea.ro (D.M.); caur@uoradea.ro (C.A.); msandor@uoradea.ro (M.S.); 3 Calla—Infertility Diagnostic and Treatment Center, Constantin A. Rosetti Street, 410103 Oradea, Romania; petronelanaghi@gmail.com; 4 Emergency County Clinical Hospital Bihor, Obstetrics-Gynecology Department,, Str. Republicii Nr. 37, 410167 Oradea, Romania; 5 Preclinical Sciences Department, Faculty of Medicine, Apollonia University, 700511 Iasi, Romania; 6 CF Oradea Clinical Hospital, Str. Republic 56, 410159 Oradea, Romania

**Keywords:** sperm DNA fragmentation, sperm morphology, teratozoospermia index, male infertility, spermatogenesis, semen analysis, assisted reproductive technology, genomic integrity

## Abstract

*Background and Objectives*: Although conventional semen analysis remains central in male infertility evaluation, the biological relationship between sperm morphology and genomic integrity remains incompletely defined. Sperm DNA fragmentation (SDF) has emerged as a clinically relevant marker of genomic instability; however, its relationship with composite morphological indices of spermatogenic dysfunction remains debated. This study aimed to evaluate the relationship between sperm DNA fragmentation assessed in the post-swim-up fraction and composite sperm morphological indices derived from raw semen, using a multivariable analytical framework that accounts for conventional semen parameters. *Materials and Methods*: This observational study included 183 semen samples from men undergoing fertility evaluation. SDF was assessed using a sperm chromatin dispersion (SCD)-based assay in the post-swim-up fraction. Sperm morphology was evaluated in raw semen according to World Health Organization criteria, and composite morphological indices, namely the Teratozoospermia Index (TZI), Sperm Deformity Index (SDI), and Multiple Anomalies Index (MAI), were calculated. Associations were examined using Spearman correlation and multivariable linear regression models adjusted for sperm concentration and progressive motility. Exploratory distributional analyses were performed across clinically defined SDF categories. *Results*: Bivariate analyses demonstrated weak, non-significant positive correlations between SDF and all composite morphological indices. None of the morphological indices independently predicted SDF after adjustment for sperm concentration and progressive motility in multivariable regression models. In contrast, sperm concentration showed a consistent inverse association with SDF. Distributional analyses revealed substantial overlap between morphological severity and SDF categories, indicating heterogeneity in the co-occurrence of structural abnormalities and DNA fragmentation at the individual sample level. *Conclusions*: Composite sperm morphological indices were not independently associated with sperm DNA fragmentation after adjusting for quantitative semen parameters in the present analytical framework. These findings suggest that structural abnormalities and genomic instability may capture complementary aspects of male infertility rather than representing interchangeable markers. SDF assessment may therefore provide complementary diagnostic information beyond morphology-based evaluation, particularly in assisted reproductive contexts.

## 1. Introduction

Despite advances in assisted reproductive technologies, male infertility remains a complex clinical and biological challenge, with conventional semen parameters often providing limited insight into the underlying causes of impaired reproductive potential [1,2]. While sperm concentration and motility describe the quantitative and functional aspects of semen, they do not fully capture the intrinsic quality of the spermatozoon as a highly specialized cell resulting from a tightly regulated process of spermatogenesis [3].

Sperm DNA fragmentation (SDF) has emerged as a critical marker of genomic integrity and male reproductive health. Elevated levels of SDF have been associated with reduced fertilization rates, impaired embryo development, increased miscarriage risk, and suboptimal outcomes in both natural conception and assisted reproduction [4,5,6]. From a biological perspective, SDF reflects alterations in chromatin remodeling, defective DNA repair mechanisms, oxidative stress, and errors occurring during meiotic and post-meiotic maturation [7,8]. Importantly, spermatozoa exhibiting normal motility and concentration may still harbor significant DNA damage, underscoring the limitations of conventional semen analysis [9].

Sperm morphology represents another fundamental dimension of sperm quality, as it reflects the structural integrity of the head, midpiece, and tail, all of which are formed during spermatogenesis. Composite morphological indices, such as the Teratozoospermia Index (TZI), Sperm Deformity Index (SDI), and Multiple Anomalies Index (MAI), provide a quantitative assessment of the severity and complexity of morphological abnormalities by capturing the number and distribution of defects within individual spermatozoa [10,11,12]. Unlike isolated morphological classifications, these indices provide a more comprehensive assessment of spermatogenic dysfunction, highlighting multiple, coexisting structural defects.

Although both SDF and sperm morphology are recognized as markers of sperm quality, their interrelationship remains incompletely understood. Existing studies have often focused on individual morphological abnormalities or have failed to account for potential confounding effects of sperm concentration and motility [13,14]. Consequently, it remains unclear whether the association between abnormal morphology and DNA fragmentation reflects a shared biological origin rooted in defective spermatogenesis or merely represents a nonspecific manifestation of poor semen quality.

Clarifying this relationship is particularly relevant in the context of intracytoplasmic sperm injection (ICSI), where sperm selection is predominantly based on morphological criteria. Structural abnormalities associated with increased DNA fragmentation may compromise embryonic development despite successful fertilization, raising concerns regarding the biological adequacy of morphology-based sperm selection alone [15,16].

The present study aimed to investigate the association between sperm DNA fragmentation and composite morphological indices (TZI, SDI, and MAI), while controlling for sperm concentration and progressive motility. By focusing on structural and genomic parameters, this study seeks to provide a more biology-oriented understanding of male infertility, emphasizing intrinsic defects of spermatogenesis rather than functional behavior in a liquid environment. In this context, the findings suggest that sperm structural complexity and genomic integrity may reflect partially distinct biological processes, as composite morphological indices were not independently associated with sperm DNA fragmentation after adjustment for conventional semen parameters. From a clinical perspective, this study explores whether baseline sperm morphology reflects the genomic integrity of the motile sperm fraction selected for use in assisted reproductive technologies.

## 2. Materials and Methods

### 2.1. Study Design and Sample Population

This observational study included 183 semen samples obtained from men referred for infertility evaluation at a tertiary reproductive medicine center (Calla IVF Center). The cohort reflects a clinically heterogeneous population undergoing routine diagnostic assessment for male factor infertility, including both primary and secondary infertility workups.

Semen samples were collected by masturbation after a standardized period of sexual abstinence of 2–7 days, in accordance with standard laboratory practice, and allowed to liquefy completely at room temperature. All samples were processed and analyzed under standardized laboratory conditions, in compliance with the World Health Organization (WHO) Laboratory Manual for the Examination and Processing of Human Semen, 6th edition [17].

The study population included a spectrum of semen profiles ranging from normozoospermia to varying degrees of quantitative and qualitative alterations in sperm concentration, motility, and morphology, thereby reflecting real-world clinical heterogeneity in an ART setting. The retrospective analysis of anonymized laboratory data was approved by the Institutional Review Board of Calla IVF Center (Approval No. 1123/A/22.02.2024). Only samples with complete data on sperm DNA fragmentation, sperm morphology, sperm concentration, and progressive motility were included in the analysis. Each semen sample corresponded to a unique individual. Only one sample per participant was included in the analysis to ensure the statistical independence of observations. Samples that did not allow adequate assessment of sperm DNA fragmentation (e.g., due to insufficient motile sperm following swim-up) were not included in the analysis.

Due to the retrospective nature of the dataset, detailed clinical and demographic variables such as age, body mass index (BMI), underlying clinical conditions (e.g., varicocele), inflammatory markers (e.g., leukocytospermia), medication or antioxidant use, and lifestyle factors (smoking and alcohol consumption) were not available and could not be included in the analysis or reported. Although abstinence duration was standardized between 2 and 7 days, the precise distribution within this range was not recorded. The study reporting follows STROBE guidelines (see Supplementary Table S2).

### 2.2. Assessment of Sperm DNA Fragmentation

Sperm DNA fragmentation (SDF) was assessed using a sperm chromatin dispersion (SCD)-based assay (halo test), which is routinely used in clinical laboratory practice. The assay was performed using a standardized in-house protocol based on the sperm chromatin dispersion (SCD) technique, following established laboratory procedures comparable to commercially available halo test kits. Following complete liquefaction, semen samples were gently homogenized, and a 200 µL aliquot from each sample was transferred into a 1.5 mL microcentrifuge tube. A swim-up procedure was performed by carefully layering 100 µL of sperm wash medium (Fertipro, Beernem, Belgium) over the sample, then incubating for 1 h to allow motile spermatozoa to migrate into the upper fraction.

After incubation, 20 µL of the supernatant was collected and mixed with 40 µL of melted agarose. An 8 µL aliquot of this mixture was placed onto a pretreated microscope slide and covered with a coverslip. Slides were refrigerated for 5 min to facilitate agarose solidification and then sequentially treated with a denaturation solution for 7 min and a lysis solution for 20 min. Slides were then rinsed with distilled water, dehydrated in graded ethanol solutions (70% and 100%), air-dried, and stained using eosin and thiazine solutions.

The prepared slides were examined under light microscopy using a dedicated image acquisition system. However, formal blinding to sperm morphology and conventional semen parameters, as well as interobserver agreement metrics, were not assessed. A minimum of 200 spermatozoa per sample was evaluated for the assessment of DNA fragmentation. Spermatozoa were classified according to halo morphology as non-fragmented (large halos equal to or greater than the sperm head diameter) or fragmented (small halos, absence of halos, or highly degraded nuclei). The percentage of spermatozoa exhibiting DNA fragmentation was calculated for each sample and expressed as SDF (%). SDF was assessed exclusively in the post-swim-up motile sperm fraction, whereas morphological evaluation and composite indices (TZI, SDI, and MAI) were determined in raw semen samples according to WHO criteria. This approach reflects routine clinical ART practice, where sperm DNA fragmentation is frequently evaluated in the motile fraction intended for fertilization, while morphology assessment provides a baseline estimate of spermatogenic structural integrity in the total ejaculate. However, this represents a fraction-specific analysis rather than a direct one-to-one comparison within the same sperm population.

SCD-based assays primarily detect specific patterns of DNA fragmentation and may underestimate the extent of DNA damage compared with other techniques, such as TUNEL. In addition, halo-based assessment is influenced by sperm chromatin structure and cell viability, which may affect the proportion of detectable DNA damage.

All procedures were performed under standardized laboratory conditions with internal quality control measures routinely applied to ensure consistency of sample processing and slide evaluation.

### 2.3. Evaluation of Sperm Morphology and Morphological Indices

Sperm morphology was evaluated according to WHO recommendations using standardized light microscopic assessment using Papanicolaou staining under oil immersion at 1000× magnification. A minimum of 200 spermatozoa per sample was assessed using systematic field scanning according to the WHO 6th edition criteria. Although morphological assessment is subject to interobserver variability, all evaluations were performed under standardized laboratory protocols and internal quality control procedures to ensure consistency of scoring. Morphological abnormalities involving the sperm head, midpiece, and tail were recorded, and composite morphological indices were calculated to quantify the severity and complexity of structural defects. Defect classification followed the WHO 6th edition criteria, including abnormalities of the head (e.g., shape, acrosome, vacuoles); midpiece (e.g., bent, irregular insertion); tail (e.g., coiled, short, multiple); and excess residual cytoplasm.

The Teratozoospermia Index (TZI) was calculated as the average number of morphological defects per abnormal spermatozoon, defined as the ratio between the total number of recorded head, midpiece, tail, and cytoplasmic defects and the number of abnormal spermatozoa evaluated. The Sperm Deformity Index (SDI) was calculated as the total number of recorded morphological defects divided by the total number of spermatozoa assessed. The Multiple Anomalies Index (MAI) was calculated as the total number of morphological defects divided by the number of abnormal spermatozoa, according to the multiple-entry recording system described in the literature. The TZI and MAI differ in their recording approaches: TZI is based on a single-entry system, in which each defect category is counted once per spermatozoon, whereas MAI uses a multiple-entry system, allowing multiple defects within the same category to be recorded. Those indices were used as quantitative markers of structural alterations in spermatogenesis.

### 2.4. Conventional Semen Parameters

In addition to SDF and morphological assessment, conventional semen parameters were recorded for all samples, including sperm concentration (million/mL) and progressive motility (%). These parameters were not considered primary variables of interest but were included as covariates to control for potential confounding effects in multivariate analyses.

### 2.5. Variables and Outcome Measures

The primary outcome variable was sperm DNA fragmentation (SDF, %), analyzed as a continuous measure of genomic instability. The main predictor variables were the composite morphological indices TZI, SDI, and MAI, reflecting the severity and complexity of sperm structural abnormalities. Sperm concentration and progressive motility were included as covariates for adjustment purposes.

### 2.6. Statistical Analysis

Statistical analyses were performed using IBM SPSS Statistics version 26.0 (IBM Corp., Armonk, NY, USA). The distribution of continuous variables was assessed using the Shapiro–Wilk test. Normally distributed variables are presented as mean ± standard deviation, whereas non-normally distributed variables are reported as median (interquartile range, IQR).

Bivariate associations between sperm DNA fragmentation (SDF) and composite morphological indices (TZI, SDI, and MAI) were evaluated using Spearman’s rank correlation, selected due to the non-normal distribution of biological variables and to assess monotonic relationships.

To evaluate independent associations, multivariable linear regression models were constructed with SDF as the dependent variable. Each morphological index was entered separately as the primary predictor to minimize collinearity. All models were adjusted for sperm concentration and progressive motility. Regression coefficients (β), 95% confidence intervals, and corresponding *p*-values were reported. Model fit was assessed using R^2^ and adjusted R^2^ values.

Multicollinearity was evaluated using variance inflation factors (VIFs), with values < 2 indicating low collinearity. The assumptions of linear regression were examined using residual plots and influence diagnostics. Residual inspection did not indicate substantial deviations from linearity or homoscedasticity, and Cook’s distance did not identify influential observations.

Given the multifactorial biological determinants of SDF, regression models were interpreted primarily to assess the independence and direction of associations rather than their predictive strength.

All statistical tests were two-tailed, and a *p*-value < 0.05 was considered statistically significant. Sensitivity analyses were conducted using alternative SDF thresholds (≥20% and ≥30%) to evaluate the robustness of the findings.

## 3. Results

**Figure 1:**
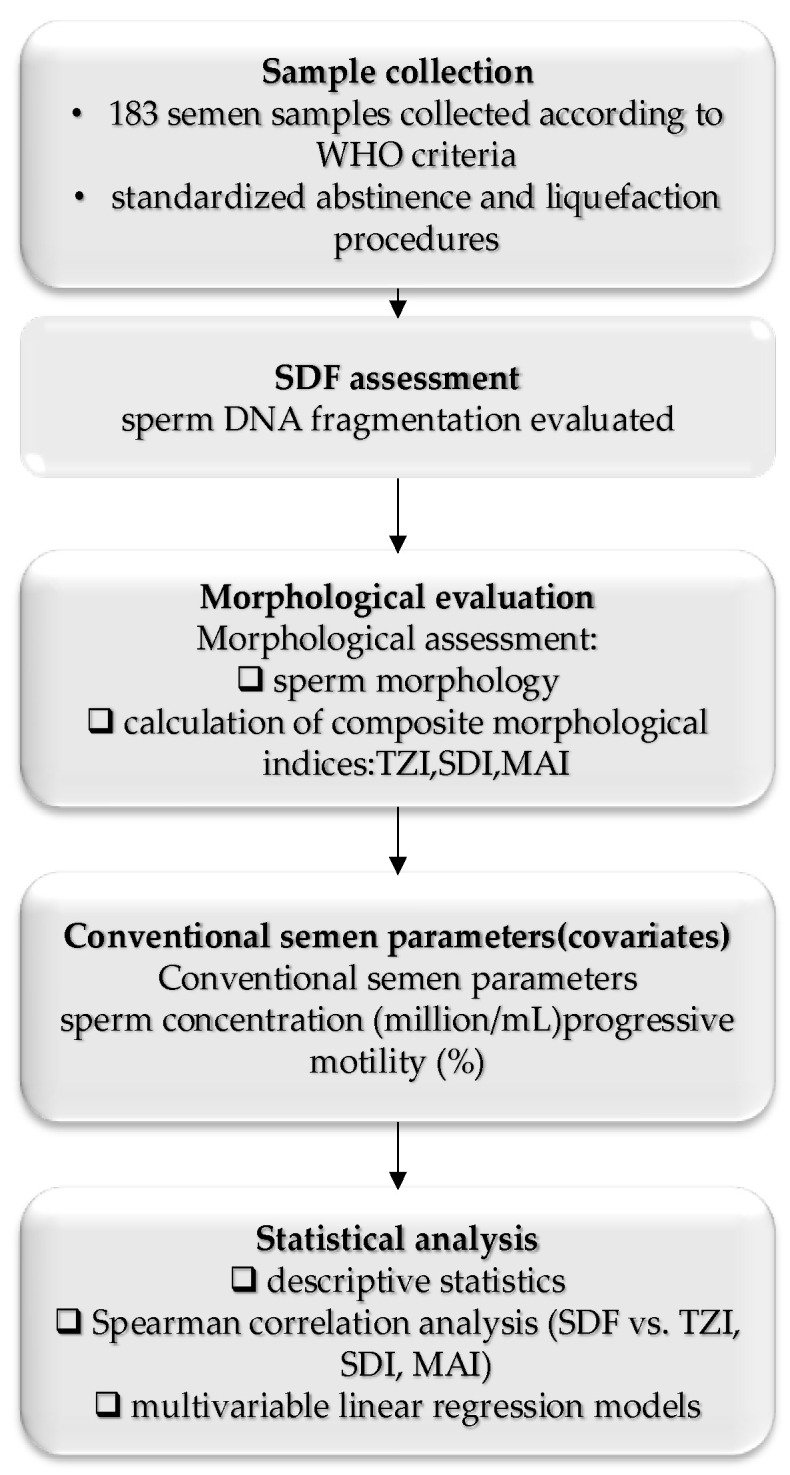
Workflow of the study evaluating the association between sperm DNA fragmentation and morphological indices, adjusted for conventional semen parameters.

Figure 1 presents the study workflow, outlining the sequential assessment of sperm DNA fragmentation and sperm morphological indices, with adjustment for conventional semen parameters to isolate morphology–SDF associations. The workflow reflects SDF assessment in the post-swim-up fraction and morphological assessment in raw semen.

### 3.1. Baseline Characteristics of the Study Population

A total of 183 semen samples were included in the final analysis. Baseline descriptive characteristics of sperm DNA fragmentation, morphological indices (TZI, SDI, and MAI), and conventional semen parameters are presented in Table 1. A more detailed descriptive analysis is provided in Supplementary Table S1. Sperm DNA fragmentation and morphological indices showed a broad distribution across the study population, indicating substantial interindividual variability in genomic integrity and sperm morphology.

Sperm concentration and progressive motility also showed wide variability and were included as covariates in subsequent analyses to control for potential confounding effects.

### 3.2. Correlation Between Sperm DNA Fragmentation and Morphological Indices

Bivariate correlation analysis showed weak positive associations between sperm DNA fragmentation (SDF) and the composite morphological indices. Spearman correlation analysis indicated a low positive correlation between SDF and the Teratozoospermia Index (TZI) (ρ = 0.053, *p* = 0.814). Similarly, SDF demonstrated weak positive correlations with the Sperm Deformity Index (SDI) (ρ = 0.085, *p* = 0.707) and the Multiple Anomalies Index (MAI) (ρ = 0.187, *p* = 0.405).

Although the correlations were consistently positive across all three indices, none of the bivariate associations were statistically significant. These findings suggest that the relationship between sperm DNA fragmentation and morphological abnormalities may not be adequately captured by unadjusted correlations, highlighting the need for multivariable modeling to account for potential confounding factors.

The Spearman correlation coefficients (ρ) and corresponding *p*-values for all bivariate analyses are summarized in Table 2.

### 3.3. Multivariable Regression Analysis

To evaluate whether sperm morphological abnormalities were independently associated with sperm DNA fragmentation, multivariable linear regression analyses were performed. In all models, sperm DNA fragmentation (SDF, %) was the dependent variable, and each composite morphological index (TZI, SDI, and MAI) was entered separately as a main predictor. Sperm concentration and progressive motility were included as covariates to control for potential confounding effects of conventional semen parameters.

Across all models, none of the composite morphological indices showed a statistically significant independent association with SDF after adjustment for sperm concentration and progressive motility. The overall explanatory power of the models was modest, with adjusted R^2^ values ranging from 0.061 to 0.094, indicating that 6–9% of the SDF variance was explained by the included predictors. Variance inflation factors (VIFs) were below 2 in all models, indicating no multicollinearity. The modest R^2^ values indicate limited explanatory and predictive performance of the models and suggest that additional unmeasured biological and clinical factors may contribute to variability in sperm DNA fragmentation. Consequently, the observed lack of independent associations should be interpreted with caution.

In contrast, sperm concentration demonstrated a consistent, statistically significant inverse association with SDF across all models, whereas progressive motility showed a negative but non-significant trend. These findings suggest that sperm DNA fragmentation in this cohort is more closely associated with quantitative semen parameters than with composite morphological indices.

The results of all multivariable regression models, including regression coefficients (β), 95% confidence intervals, and *p*-values, are summarized in Table 3.

Because SDF thresholds are assay-dependent and not universally defined by WHO, sensitivity analyses were performed using alternative cut-offs commonly reported in SCD-based studies. Sensitivity analyses using alternative SDF thresholds showed that 111/183 (60.7%) samples had SDF ≥ 20%, and 47/183 (25.7%) had SDF ≥ 30%. Reclassification using these cut-offs did not materially alter the direction or strength of associations with morphological indices.

### 3.4. Distribution of Morphological Severity Across SDF Categories

To further explore the relationship between sperm structural abnormalities and genomic integrity, a categorical distributional analysis was performed across clinically defined SDF strata.

Samples were grouped into four SDF categories (1–15%, 15.01–25%, 25.01–50%, and >50%), and morphological severity was dichotomized using the cohort median value of the Multiple Anomalies Index (MAI = 2.84) for descriptive comparison.

The cross-tabulation revealed heterogeneous structural profiles across SDF categories. Elevated MAI values were observed in both mild and moderate SDF groups, while a proportion of samples with higher SDF levels exhibited lower MAI values.

A chi-square test demonstrated a statistically significant association between SDF category and MAI classification (χ^2^ (3) = 35.39, *p* < 0.001; Cramér’s V = 0.44). However, this categorical association reflects grouped distribution patterns and does not imply a consistent linear relationship between continuous morphological indices and SDF values at the individual level.

Consistent with the regression analyses presented in Section 3.3, composite morphological indices did not demonstrate independent predictive capacity for SDF when analyzed as continuous variables adjusted for conventional semen parameters. As summarized in Table 4, these results indicate that sperm structural abnormalities do not consistently coincide with higher levels of genomic damage. While a categorical association was observed, the findings do not support a consistent linear concordance between morphology and SDF at the individual sample level.

## 4. Discussion

The present study examined the relationship between sperm DNA fragmentation (SDF) and composite morphological indices (TZI, SDI, and MAI) to determine whether structural abnormalities in spermatozoa independently reflect underlying genomic instability. By integrating bivariate correlation analysis, multivariable regression modeling, and categorical distributional assessment across predefined SDF strata, this study provides a structured evaluation of the interplay between sperm morphology and DNA integrity.

Although weak positive correlations between SDF and TZI, SDI, and MAI were observed at the bivariate level, these associations were small in magnitude and did not persist after adjustment for sperm concentration and progressive motility. In multivariable models, composite morphological indices did not independently explain variability in SDF within the present study’s methodological framework. These findings suggest that composite morphological indices do not provide additional independent information on sperm DNA fragmentation beyond conventional semen parameters such as sperm concentration and progressive motility. The modest adjusted R^2^ values further indicate limited predictive performance, suggesting that additional biological determinants contribute to SDF variability beyond structural morphology alone. These findings indicate that increasing morphological complexity does not translate into a proportional or linear increase in DNA fragmentation when conventional semen parameters are considered.

These results are consistent with accumulating evidence suggesting that sperm morphology and DNA integrity may reflect partially distinct aspects of spermatogenic function [18,19]. Although both parameters originate in spermatogenesis, they likely reflect distinct biological mechanisms and stages within this process. Morphological abnormalities arise primarily from disruptions in cytoskeletal organization, acrosome formation, manchette function, and flagellar assembly, whereas DNA fragmentation is more closely linked to defective chromatin remodeling, impaired protamination, oxidative stress, and abortive apoptosis [20,21,22]. Consequently, structural and genomic alterations may coexist in some individuals while diverging in others, depending on the dominant pathogenic pathway.

The categorical analysis across SDF strata revealed a statistically significant association between SDF category and MAI classification; however, this finding reflects distributional clustering rather than a stable linear predictive relationship. Severe morphological profiles were not restricted to samples with elevated SDF, and conversely, some samples with high SDF exhibited non-severe morphological patterns. This overlap underscores the heterogeneity of male infertility phenotypes and highlights the limitations of inferring genomic integrity solely from individual-level morphological assessment.

From a clinical perspective, these observations are particularly relevant in assisted reproductive technology (ART). Thus, the findings should be interpreted primarily in a clinical context, rather than as a direct biological comparison within the same sperm population. In intracytoplasmic sperm injection (ICSI), sperm selection is primarily based on morphological evaluation under light microscopy. However, previous studies have demonstrated that morphologically normal spermatozoa may harbor significant DNA damage, potentially affecting embryo development and pregnancy outcomes [23,24,25]. The present findings support the notion that morphology alone may not fully capture genomic quality, reinforcing the rationale for independent SDF assessment in selected clinical contexts.

Interestingly, sperm concentration was the only parameter independently associated with SDF in adjusted models, displaying a consistent inverse relationship. This association is biologically plausible and aligns with previous evidence linking oligozoospermia to oxidative stress, impaired chromatin packaging, and defective spermatogenesis [26,27,28].

Sperm concentration and progressive motility were included as adjustment variables to account for overall semen quality. However, these parameters may also reflect shared biological pathways with morphology and DNA fragmentation and therefore may partially act as intermediate variables rather than purely independent confounders. This should be considered when interpreting the adjusted associations.

In contrast, progressive motility did not independently correlate with SDF, suggesting that functional motility in a liquid medium does not necessarily reflect underlying chromatin integrity in heterogeneous clinical populations.

Several limitations should be acknowledged. The cross-sectional design precludes causal inference. SDF was assessed using a single SCD-based assay, which is not interchangeable with other methodologies, such as TUNEL or SCSA, and may capture specific aspects of DNA damage. Regression models were adjusted only for sperm concentration and progressive motility because additional clinical and lifestyle variables were unavailable in the retrospective dataset; therefore, residual confounding cannot be excluded. Important factors such as age, lifestyle behaviors, and underlying clinical conditions were not accounted for and may have influenced the strength and direction of the observed associations.

Importantly, SDF was assessed in the post-swim-up motile fraction, whereas morphology was evaluated in raw semen samples, which introduces a potential selection-related bias and limits direct biological comparability between the analyzed parameters. This fraction-specific assessment reflects routine ART practice, where genomic integrity is evaluated in the motile sperm population used for fertilization, while morphology provides a baseline estimate of structural integrity in the total ejaculate.

However, the post-swim-up fraction is enriched in motile and functionally competent spermatozoa, which may differ substantially from the heterogeneous population present in raw semen. Moreover, sperm selection techniques such as swim-up are known to reduce DNA fragmentation levels compared to the raw ejaculate, which may decrease SDF variability and potentially attenuate or mask underlying associations between sperm morphology and genomic integrity. Consequently, this methodological discrepancy may have attenuated potential associations between morphological indices and SDF and could partly explain the lack of independent relationships observed in the present study.

Although abstinence duration was standardized according to WHO recommendations (2–7 days), individual variability within this range could not be modeled as a covariate.

An additional limitation should be considered. The present study did not include embryological or clinical outcomes such as fertilization rate, embryo development, embryo quality, or pregnancy outcomes. Therefore, the clinical relevance of the observed lack of association between sperm morphology and DNA fragmentation cannot be directly assessed. Although both parameters are known to influence reproductive success, the absence of outcome-based validation limits the ability to translate these findings into clinical decision-making, particularly in the context of assisted reproductive technologies. Future studies integrating molecular sperm parameters with fertilization, embryo development, and pregnancy outcomes are warranted to better define their clinical significance.

Although SDF was analyzed as a continuous outcome, it represents a bounded proportional variable; therefore, alternative modeling approaches (e.g., fractional response models) could be considered in future studies as robustness analyses.

Despite these limitations, the study benefits from a comprehensive analytical framework that integrates continuous and categorical approaches, provides explicit regression diagnostics, and conducts sensitivity analyses using alternative SDF thresholds. The distinction between group-level association and individual-level prediction offers a more nuanced interpretation of the morphology–genome interplay.

Overall, the findings indicate that composite sperm morphological indices do not independently predict sperm DNA fragmentation in this cohort. Rather than representing interchangeable markers, morphology and genomic integrity appear to capture complementary aspects of sperm quality. These results support the consideration of SDF testing as an adjunctive tool in selected clinical scenarios, particularly within assisted reproduction, while emphasizing the need for prospective studies integrating molecular markers with fertilization, embryo development, and pregnancy outcomes. These findings should be interpreted with caution, given the limited explanatory power of the models.

## 5. Conclusions

In this cohort, composite sperm morphological indices, namely the Teratozoospermia Index (TZI), Sperm Deformity Index (SDI), and Multiple Anomalies Index (MAI), did not provide additional independent information on sperm DNA fragmentation beyond conventional semen parameters. Although morphological abnormalities and elevated SDF may coexist within the same sample, their relationship does not appear to follow a consistent linear pattern when analyzed as continuous variables.

The inverse association observed between sperm concentration and SDF supports the contribution of impaired spermatogenesis and cellular stress to genomic instability. In contrast, progressive motility did not independently predict SDF within the present analytical framework, suggesting a limited ability of conventional functional parameters to reliably infer chromatin integrity in heterogeneous clinical cohorts. From a clinical standpoint, these findings suggest that sperm morphology alone may not adequately reflect genomic integrity. Rather than serving as interchangeable indicators, structural and molecular markers appear to provide complementary information regarding sperm quality. The integration of sperm DNA fragmentation testing with conventional semen analysis may therefore refine diagnosis in selected clinical scenarios, particularly within assisted reproductive technologies. However, prospective studies incorporating reproductive outcomes are required to determine the true clinical utility of combined structural and genomic assessment.

## Figures and Tables

**Table 1:** Baseline semen parameters and sperm DNA fragmentation distribution in the study cohort (*n* = 183).

Variable	Value
Sperm DNA fragmentation (SDF, %)	22.43 (16.69–30.50)
SDF category—control (1–15%)	22 (12.0%)
SDF category—mild (15.01–25%)	99 (54.1%)
SDF category—moderate (25.01–50%)	53 (29.0%)
SDF category—severe (>50%)	9 (4.9%)
Sperm concentration (million/mL)	36.95 ± 23.41
Progressive motility (%)	53.22 ± 18.48
Teratozoospermia Index (TZI)	1.29 ± 0.11
Sperm Deformity Index (SDI)	1.99 ± 0.22
Multiple Anomalies Index (MAI)	2.84 (2.40–3.14)

**Table 2:** Spearman correlation coefficients (ρ) and *p*-values between sperm DNA fragmentation and morphological indices.

Correlation	Spearman Coefficient (ρ)	*p*-Value
SDF–TZI	0.053	0.814
SDF–SDI	0.085	0.707
SDF–MAI	0.187	0.405

**Table 3:** Multivariable linear regression analysis evaluating the association between sperm DNA fragmentation and morphological indices.

Predictor	TZI Model β (95% CI)	*p*-Value	SDI Model β (95% CI)	*p*-Value	MAI Model β (95% CI)	*p*-Value
Morphological index	−1.14 (−11.45 to 9.16)	0.818	−0.95 (−5.37 to 3.48)	0.659	−0.69 (−5.68 to 4.29)	0.773
Sperm concentration (million/mL)	−0.047 (−0.092 to −0.001)	0.046	−0.050 (−0.092 to −0.009)	0.020	−0.050 (−0.093 to −0.008)	0.022
Progressive motility (%)	−0.044 (−0.100 to 0.011)	0.112	−0.044 (−0.097 to 0.009)	0.095	−0.042 (−0.094 to 0.010)	0.104

Note: Each column represents a separate multivariable linear regression model. Sperm DNA fragmentation (%) was included as the dependent variable. TZI, SDI, and MAI were entered individually as predictors to avoid collinearity. All models were adjusted for sperm concentration and progressive motility.

**Table 4:** Distribution of morphological severity across predefined SDF categories (MAI cut-off = 2.84, cohort median).

SDF Category	*n*	Low MAI (<2.84)	High MAI (≥2.84)
Control (1–15%)	22	22 (100.0%)	0 (0.0%)
Mild (15.01–25%)	99	33 (33.3%)	66 (66.7%)
Moderate (25.01–50%)	53	32 (60.4%)	21 (39.6%)
Severe (>50%)	9	4 (44.4%)	5 (55.6%)
Total	183	91 (49.7%)	92 (50.3%)

Note: Morphological classification was based on the Multiple Anomalies Index (MAI). Samples were categorized as low or high MAI using the cohort median value (MAI = 2.84). SDF was grouped into four predefined categories for descriptive comparison. Values are presented as *n* (% within each SDF category). A chi-square test was used to assess the association between SDF category and MAI classification (χ^2^ (3) = 35.39, *p* < 0.001; Cramér’s V = 0.44).

## Data Availability

The original contributions presented in this study are included in the article. For further inquiries, please contact the corresponding authors.

## Supplementary Materials

The following supporting information can be downloaded at: https://www.mdpi.com/article/10.3390/medicina62040679/s1, Supplementary Table S1. Data Audit of SDF and Morphological Indices (n = 183), Supplementary Table S2. STROBE Checklist (Observational Study).
