# Supplementary material for: Relationship Between Sperm DNA Fragmentation and Composite Morphological Indices: A Multivariable Analysis
Source: Medicina (Kaunas). 2026 Apr 2;62(4):679. doi: 10.3390/medicina62040679 (PMC13118042; doi:10.3390/medicina62040679)
Supplement: Supplementary file 1 [file medicina-62-00679-s001.zip › medicina-4198201-supplementary.pdf]

Supplementary Table S1. Data Audit of SDF and Morphological Indices (n = 183)

Variable	n	Min	Max	Median (IQR)	Mean ± SD
Sperm DNA Fragmentation (SDF, %)	183	1.00	70.00	22.43 (16.69–30.50)	—
Multiple Anomalies Index (MAI)	183	1.90	3.80	2.84 (2.40–3.14)	2.78 ± 0.53
Teratozoospermia Index (TZI)	183	1.00	1.60	1.29 (1.22–1.36)	1.29 ± 0.11
Sperm Deformity Index (SDI)	183	1.50	2.50	1.99 (1.85–2.12)	1.99 ± 0.22
MAI < 2.84 (Low MAI)	91	—	—	—	49.7% of cohort
MAI ≥ 2.84 (High MAI)	92	—	—	—	50.3% of cohort

Supplementary Table S2. STROBE Checklist (Observational Study)

STROBE Item	Recommendation	Location in Manuscript
1. Title and abstract	Indicate study design in title/abstract	Abstract – study described as a retrospective observational study
2. Background/rationale	Explain the scientific background and rationale	Introduction, paragraphs 1–3
3. Objectives	State-specific objectives and hypotheses	End of Introduction
4. Study design	Present key design elements early	Section 2.1 Study Design and Sample Population
5. Setting	Describe setting, location, dates	Section 2.1 (Calla IVF Center, retrospective laboratory data)
6. Participants	Eligibility criteria and selection methods	Section 2.1 (Inclusion criteria; complete datasets only)
7. Variables	Define outcomes, exposures, and confounders	Sections 2.1–2.3 and 2.6

 https://doi.org/10.3390/medicina62040679

8. Data sources/measurement	Describe measurement methods	Section 2.1 (WHO 6th edition; swim-up vs raw semen clarification)
9. Bias	Describe efforts to address potential bias	Discussion – Limitations section
10. Study size	Explain how the study size was determined	Section 2.1 (All eligible samples; n = 183)
11. Quantitative variables	Explain the handling and categorization of quantitative variables	Section 2.6 (Median/IQR, regression, MAI cut-off 2.84)
12. Statistical methods	Describe statistical methods, including confounder control	Section 2.6 Statistical Analysis
13. Participants (Results)	Report numbers at each stage	Section 3.1 (Total included samples)
14. Descriptive data	Present participant characteristics	Table 1
15. Outcome data	Report outcome measures	Table 1: Results section 3
16. Main results	Provide unadjusted and adjusted estimates	Sections 3.2–3.3
17. Other analyses	Report sensitivity analyses	Results – Sensitivity analysis paragraph
18. Key results (Discussion)	Summarize key findings	Discussion – first paragraph
19. Limitations	Discuss study limitations and bias	Discussion – Limitations paragraph
20. Interpretation	Interpret results in context	Discussion main body
21. Generalizability	Discuss external validity	Discussion – final paragraph
22. Funding	Report funding sources and role of funders	Funding statement

https://doi.org/10.3390/medicina62040679
